# Survival Benefits of Statins for Primary Prevention: A Cohort Study

**DOI:** 10.1371/journal.pone.0166847

**Published:** 2016-11-18

**Authors:** Lisanne A. Gitsels, Elena Kulinskaya, Nicholas Steel

**Affiliations:** 1 School of Computing Sciences, University of East Anglia, Norwich, United Kingdom; 2 Norwich Medical School, University of East Anglia, Norwich, United Kingdom; University of British Columbia, CANADA

## Abstract

**Objectives:**

Estimate the effect of statin prescription on mortality in the population of England and Wales with no previous history of cardiovascular disease.

**Methods:**

Primary care records from The Health Improvement Network 1987–2011 were used. Four cohorts of participants aged 60, 65, 70, or 75 years at baseline included 118,700, 199,574, 247,149, and 194,085 participants; and 1.4, 1.9, 1.8, and 1.1 million person-years of data, respectively. The exposure was any statin prescription at any time before the participant reached the baseline age (60, 65, 70 or 75) and the outcome was all-cause mortality at any age above the baseline age. The hazard of mortality associated with statin prescription was calculated by Cox’s proportional hazard regressions, adjusted for sex, year of birth, socioeconomic status, diabetes, antihypertensive medication, hypercholesterolaemia, body mass index, smoking status, and general practice. Participants were grouped by QRISK2 baseline risk of a first cardiovascular event in the next ten years of <10%, 10–19%, or ≥20%.

**Results:**

There was no reduction in all-cause mortality for statin prescription initiated in participants with a QRISK2 score <10% at any baseline age, or in participants aged 60 at baseline in any risk group. Mortality was lower in participants with a QRISK2 score ≥20% if statin prescription had been initiated by age 65 (adjusted hazard ratio (HR) 0.86 (0.79–0.94)), 70 (HR 0.83 (0.79–0.88)), or 75 (HR 0.82 (0.79–0.86)). Mortality reduction was uncertain with a QRISK2 score of 10–19%: the HR was 1.00 (0.91–1.11) for statin prescription by age 65, 0.89 (0.81–0.99) by age 70, or 0.79 (0.52–1.19) by age 75.

**Conclusions:**

The current internationally recommended thresholds for statin therapy for primary prevention of cardiovascular disease in routine practice may be too low and may lead to overtreatment of younger people and those at low risk.

## Introduction

Cardiovascular disease is one of the main causes of death, accounting for 28% of all deaths in the United Kingdom [1–3]. Statins have been widely prescribed for primary and secondary prevention of cardiovascular disease since the Scandinavian Simvastatin Survival Study in the 1990s demonstrated benefits of statin therapy in patients with established cardiovascular disease (CVD [4]. Since then the results of many statin trials have been combined into an individual patient-based meta-analysis of 27 randomised control trials and over 90,000 patients, the Cholesterol Treatment Trialists’ (CTT) Collaboration [5]. The meta-analysis reported that in participants without a history of vascular disease, statins reduced the overall risk of all-cause mortality by 9% per 1.0 mmol/L reduction in LDL. The study, however, could not conclude a survival benefit by statin for the individual risk groups due to the small number of events.

In July 2014, the UK National Institute for Health and Clinical Excellence (NICE) lowered the QRISK2 estimated risk of a first cardiovascular event in the next ten years at which it recommended statins should be prescribed from 20% to 10% [6], causing a ‘storm of controversy’ about the benefits to people at low risk [7]. This translated to an increasing number of people being eligible for the drugs; that is an additional 4.5 million UK residents [8]. NICE also recommended that ‘people older than 40 should have their estimate of CVD risk reviewed on an ongoing basis’, although there is no evidence of benefit for patients in their 40s or 50s with no history of cardiovascular disease. The risk threshold of 10% recommended by NICE identifies similar numbers of patients to the 2013 American College of Cardiology/American Heart Association (ACC/AHA) guideline, which recommends for statin prescription when the Pooled Cohort Equations (PCE) estimated 10-year risk of a cardiovascular event is ≥7.5% [9–10]. The 2012 European Society of Cardiology (ESC) guideline recommends considering statins when the Systematic COronary Risk Evaluation (SCORE) estimated 10-year risk of cardiovascular mortality is ≥5%, but this identifies many fewer patient than the NICE and ACC/AHA guidelines because it focuses on mortality rather than events [9,11].

The CTT meta-analysis was one of the most comprehensive sources of evidence assembled for any medical condition, but still left some major uncertainties about the benefits of statins for those without a history of vascular disease [5]. Firstly, the strict inclusion criteria of most of the included clinical trials makes it difficult to apply the findings to patients in clinical practice, most of whom would not have been eligible for the trials on the grounds of age or morbidity [12–15]. Secondly, the risk groups were based on the observed annual major vascular events rate in the control groups, which makes comparison with the QRISK2, SCORE, or PCE risk over 10 years, as widely used and recommended in European or American clinical practice, difficult and uncertain. Thirdly, the average age of a trial participant was 63 years and the trials only included a small number of older patients, making estimates of effectiveness in different age groups difficult. Fourthly, the follow-up time of each trial was at most five years, which is very much shorter than many patients in routine clinical practice. Fifthly, submission of trial data to the CTT study was optional, and so not all clinical trials were included in the meta-analysis. There are also concerns that anonymised individual patient data from statins trials have not been made available for independent scrutiny, particularly as statins are among the most widely prescribed drugs globally [7].

NICE identified several gaps in the research evidence around statins when the guidance was updated in 2014, and recommended further research into the effectiveness of age alone and other routinely available risk factors compared with the formal structured multi-factorial risk assessment to identify people at high risk of developing CVD, and into the effectiveness of statin therapy in older people [6]. This study aimed to estimate the longer-term survival benefit from statin prescription in the general population with no previous history of cardiovascular disease, stratified by age and QRISK2 groups.

## Methods

### Study design

This study was approved by the THIN Scientific Review Committee on the 16th of June 2014 (Reference Number 14–043). A cohort study was designed on routinely collected data of primary care patient records from The Health Improvement Network (THIN) UK database (http://www.thin-uk.net/). The database currently holds 12 million patient records from 578 general practices, corresponding to 6% of the UK population, with slightly more THIN patients living in affluent areas compared to nationally [16]. THIN is generalizable to the UK population with regards to demographics, prevalence of medical conditions, and mortality rates, adjusted for deprivation [17–18].

Medical records from 1987 to 2011 of people who were born between 1920 and 1940 were selected. Four age cohorts were created that consisted of participants who turned the target age between 1^st^ January 1987 and 2011, and participants could be part of multiple age cohorts. The target ages were 60, 65, 70, and 75 in order to facilitate individuals and general practitioners to make a decision about statin uptake at key ages. Before age 60, the initiation rate of statin prescription for primary prevention of cardiovascular disease in the UK is less than 3% [19].

Participants were selected if at the target age they had been registered for at least one year at an active general practice that coded death dates validly, and their medical records had been accessed at least once within the last ten years. Participants with a previous history of cardiovascular disease (coronary artery disease, cerebrovascular disease, or peripheral vascular disease) were excluded from the analysis, as were participants living in Northern Ireland and Scotland due to unavailable Townsend deprivation scores. Participant selection was performed in SQL server 2012. Participants were followed up until the 18^th^ of March 2011, resulting in a study period of 24, 24, 21, and 16 years for the 60-, 65-, 70-, and 75-year old cohorts, respectively. Participants were lost to follow-up when they transferred to another general practice during the observation time. It was assumed that this was independent from survival time.

### Risk assessment of cardiovascular disease

The NICE guideline on lipid modification recommends using QRISK2 to calculate the 10-year risk of a first cardiovascular event [6]. The QRISK2 algorithm is available online (http://svn.clinrisk.co.uk/opensource/qrisk2/). It incorporates information on multiple demographic, medical, and lifestyle factors to estimate the risk (S1 Table) [20]. Not all this information was available or complete in THIN, leading to a number of substitutions or exclusions. The QRISK2 was calculated for all participants with complete information in JAVA version 8.

Ethnicity was not recorded in THIN and could therefore not be included in the QRISK2 calculation. The QRISK2 calculator has white ethnicity as the reference category. 93% of the UK population is white and the QRISK2 score risk would be underestimated for people with an Indian, Pakistani, or Bangladeshi background and overestimated for people with a black Caribbean background and for black African and Chinese men [20]. In THIN the Townsend score was available in quintiles based on the 2001 census data, and the associated median value for each quintile was used for calculating the cardiovascular risk [21]. The prevalence of atrial fibrillation and rheumatoid arthritis is <1% in the United Kingdom, making their effect on QRISK2 minimal at population level [20,22], and it was assumed that the selected participants did not have these medical conditions. Family history of cardiovascular disease is not systematically recorded in primary care, and was <1% in the four cohorts in THIN [20]. It was assumed that diabetes was type two, which would be true for 90% of the cases [23]. It was also assumed that current smokers smoked moderately [24]. The diagnosis of hypercholesterolaemia was used instead of a specific cholesterol level, because it was only recorded in the minority of primary care records (27–50% recordings) [20]. When a participant did not have a diagnosis of hypercholesterolaemia, a ratio of total cholesterol to high density lipids of four was ascribed. This is the default value of the online QRISK2 calculator when no ratio is given. When a participant had a diagnosis of hypercholesterolaemia, a ratio value of five was ascribed.

### Statistical analyses

Cox’s proportional hazard regression was used to estimate the effect of statin prescription on the hazard of mortality for different risk groups at various ages, with the outcome variable being time to death in days. All-cause mortality was used because other studies have shown the protective effect of statins on the risk of cardiovascular and cerebrovascular events and deaths, but it was still unclear what the overall survival effect is. All-cause mortality is not only an important outcome for patients, but also for the national economy. Life expectancy directly affects taxation, national insurance rates, retirement planning, including state and private pensions, and actuarial products such as life insurance and annuities. Furthermore, death is an unambiguous hard outcome that is not subject to medical classifications such as coronary revascularisation procedures, that depend on rates of diagnosis that are subject to differing professional opinions and geographic variations [25]. The exposure was any statin prescription at any time before the participant reached the baseline age. The analysis on statins excluded participants who were prescribed other lipid-lowering therapy to ensure that the effect of statins was not mediated by those drugs. The number of participants excluded at age 60, 65, 70, and 75 were 876 (0.7%), 1,718 (0.9%), 2,840 (1.1%), and 2,475 (1.3%), respectively. The analysis performed was on an intention to treat basis as it was based on the prescription of drugs, and not intake of them. The models were fitted to each age and risk group. The ages were 60, 65, 70, and 75 years. The risk groups consisted of participants with a QRISK2 of <10%, 10–19%, or ≥20% risk of a first cardiovascular event in the next ten years.

Potential confounders of the effect of statin prescription on survival were selected on the basis of expert knowledge and literature review (S2 and S3 Tables). With backward elimination, the variables that were significant (p<0.05) in the majority of the models, including interactions with statin prescription, were adjusted for in the final models. The final models adjusted for sex, year of birth, socioeconomic status, diabetes, hypercholesterolaemia, blood pressure regulating drugs, body mass index, smoking status, and general practice. Socioeconomic status was measured by Mosaic, a classification that captures demographics, lifestyles and behaviour of people at postcode level [26]. In the preliminary results, Mosaic was found to be a stronger predictor of mortality than Index of Multiple Deprivation (IMD) and Townsend deprivation score. There were missing values in systolic blood pressure (15–28%), smoking status (14–30%), and body mass index (22–37%). The fraction of incomplete medical records decreased with age; 46% of the youngest cohort and 29% of the oldest cohort had incomplete records. The proportion of complete records was greater among participants born at a later year, with a medical condition, or who were prescribed drugs (S4 Table). This is in agreement with previous research that found that recording is likely be related to ill health and that recording improved after the introduction of the Quality and Outcomes Framework (QOF) in 2004 [27–29]. Incomplete medical records were dealt with by multiple imputation (S5 Table) [30]. The distribution of known and imputed values for systolic blood pressure, body mass index, smoking status, and QRISK2 scores were similar (S6 Table).

The survival models were multilevel on general practice and participant level, to deal with the interdependence of participants from the same general practice [30]. Efron’s approximation was used to deal with time-tied deaths [31]. The number of years gained or lost due to statin prescription were calculated [32]. The models were checked for proportional hazards assumption, overall performance, discrimination, and external validation [33–36]. The sensitivity analysis included comparing the adjusted effects estimated on the complete and incomplete records with (1) the unadjusted effects estimated on the complete and incomplete records, and (2) the adjusted effects estimated on the complete records. The analysis was executed in R version 3.1.1 using the packages ‘survival’ [31] and ‘rms’ [36].

For more information on the multiple imputation, model assumptions, and model assessments, please see S5 Table.

## Results

**Fig 1:**
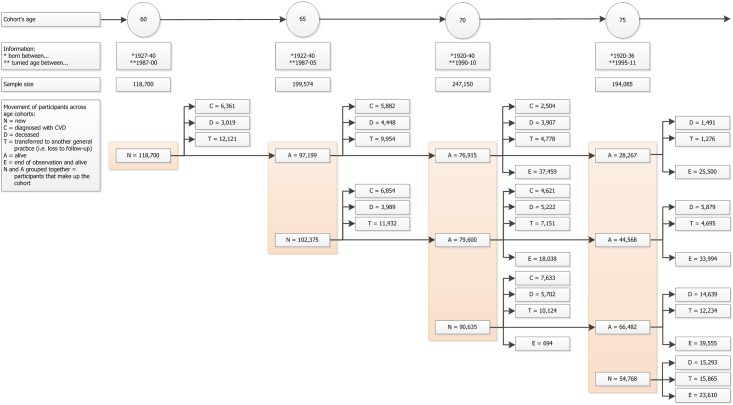
Selection age cohorts. Four cohorts born between 1920 and 1940 who reached the age of 60, 65, 70, or 75 years between 1987 and 2011 with no previous history of cardiovascular disease.

The four cohorts consisted of participants aged 60, 65, 70, and 75 years old at baseline, respectively, with no history of cardiovascular disease (Fig 1 and Tables 1–4).

**Table 1:** Baseline characteristics of the 60-year old cohort.

	QRISK2<10%		QRISK2 = 10–19%		QRISK2≥20%	
	No LLT^a^	Statins	No LLT	Statins	No LLT	Statins
Total	59,257	645	52,907	964	3,787	264
Women (%)	50,064 (84%)	604 (94%)	9,461 (18%)	373 (39%)	607 (16%)	85 (32%)
Men (%)	9,193 (16%)	41 (6%)	43,446 (82%)	591 (61%)	3,180 (84%)	179 (68%)
Born 1936–40 (%)	37,497 (63%)	560 (87%)	33,050 (62%)	873 (91%)	2,603 (69%)	252 (95%)
Born 1927–35 (%)	21,760 (37%)	85 (13%)	19,857 (38%)	91 (9%)	1,184 (31%)	12 (5%)
Townsend score • 1 least deprived (%)	22,164 (37%)	262 (41%)	14,105 (27%)	292 (30%)	698 (18%)	41 (16%)
• 2 (%)	15,829 (27%)	179 (28%)	11,967 (23%)	238 (25%)	687 (18%)	64 (24%)
• 3 (%)	10,970 (19%)	111 (17%)	10,270 (19%)	190 (20%)	733 (19%)	49 (19%)
• 4 (%)	7,404 (12%)	64 (10%)	9,525 (18%)	150 (16%)	856 (23%)	63 (24%)
• 5 most deprived (%)	2,890 (5%)	29 (4%)	7,040 (13%)	94 (10%)	813 (21%)	47 (18%)
Family history of cardiovascular disease (%)	42 (0%)	2 (0%)	63 (0%)	2 (0%)	20 (1%)	5 (2%)
Chronic kidney disease (%)	1 (0%)	0 (0%)	2 (0%)	0 (0%)	2 (0%)	0 (0%)
Diabetes (%)	17 (0%)	2 (0%)	1,563 (3%)	99 (10%)	2,220 (59%)	173 (66%)
Hypercholesterolaemia (%)	5,189 (9%)	425 (66%)	7,745 (15%)	692 (72%)	1,568 (41%)	208 (79%)
Treated hypertension (%)	6,040 (10%)	239 (37%)	10,191 (19%)	481 (50%)	2,293 (61%)	191 (72%)
Systolic blood pressure (sd)^b^	135 (17)	135 (16)	141 (18)	144 (18)	154 (20)	151 (18)
Ex-smoker (%)^b^	5,817 (10%)	77 (12%)	8,517 (16%)	202 (21%)	555 (15%)	56 (21%)
Smoker (%)^b^	7,837 (13%)	36 (6%)	20,519 (39%)	252 (26%)	2,243 (59%)	138 (52%)
Body mass index (sd)^b^	26 (4)	27 (4)	26 (4)	27 (4)	29 (5)	30 (6)

The number of participants in each risk-treatment group is the mean across ten imputed datasets.

^a^ lipid-lowering drugs ^b^ mean values across ten imputed datasets.

**Table 2:** Baseline characteristics of the 65-year old cohort.

	QRISK2<10%		QRISK2 = 10–19%		QRISK2≥20%	
	No LLT^a^	Statins	No LLT	Statins	No LLT	Statins
Total	39,866	883	116,240	6,438	29,170	5,259
Women (%)	39,866 (100%)	883 (100%)	54,094 (47%)	4,381 (68%)	4,532 (16%)	1,742 (33%)
Men (%)	0 (0%)	0 (0%)	62,146 (53%)	2,057 (32%)	24,638 (84%)	3,517 (67%)
Born 1936–40 (%)	17,901 (45%)	698 (79%)	52,206 (45%)	5,155 (80%)	13,986 (48%)	4,620 (88%)
Born 1931–35 (%)	13,804 (35%)	165 (19%)	38,748 (33%)	1,162 (18%)	9,811 (34%)	601 (11%)
Born 1922–30 (%)	8,161 (20%)	20 (2%)	25,286 (22%)	121 (2%)	5,373 (18%)	38 (1%)
Townsend score • 1 least deprived (%)	17,012 (43%)	403 (46%)	34,654 (30%)	2,168 (34%)	5,933 (20%)	1,270 (24%)
• 2 (%)	11,644 (29%)	245 (28%)	28,164 (24%)	1,673 (26%)	5,538 (19%)	1,125 (21%)
• 3 (%)	7,232 (18%)	146 (17%)	23,302 (20%)	1,208 (19%)	5,557 (19%)	1,066 (20%)
• 4 (%)	3,500 (9%)	84 (10%)	19,150 (16%)	902 (14%)	6,079 (21%)	1,043 (20%)
• 5 most deprived (%)	478 (1%)	5 (1%)	10,970 (9%)	487 (8%)	6,063 (21%)	755 (14%)
Family history of cardiovascular disease (%)	14 (0%)	0 (0%)	273 (0%)	41 (1%)	173 (1%)	51 (1%)
Chronic kidney disease (%)	3 (0%)	0 (0%)	32 (0%)	9 (0%)	15 (0%)	18 (0%)
Diabetes (%)	0 (0%)	0 (0%)	828 (1%)	439 (7%)	6,419 (22%)	3,108 (59%)
Hypercholesterolaemia (%)	3,221 (8%)	475 (54%)	22,230 (19%)	4,013 (62%)	10,565 (36%)	3,163 (60%)
Treated hypertension (%)	459 (1%)	46 (5%)	27,954 (24%)	3,616 (56%)	14,264 (49%)	4,068 (77%)
Systolic blood pressure (sd)^b^	133 (15)	127 (13)	142 (18)	140 (16)	149 (19)	146 (17)
Ex-smoker (%)^b^	4,009 (10%)	86 (10%)	23,958 (21%)	1,543 (24%)	5,446 (19%)	1,673 (32%)
Smoker (%)^b^	1,260 (3%)	4 (0%)	23,696 (20%)	612 (10%)	16,489 (57%)	1,659 (32%)
Body mass index (sd)^b^	26 (4)	26 (4)	26 (4)	28 (5)	27 (5)	29 (5)

The number of participants in each risk-treatment group is the mean across ten imputed datasets.

^a^ lipid-lowering drugs ^b^ mean values across ten imputed datasets.

**Table 3:** Baseline characteristics of the 70-year old cohort.

	QRISK2<10%		QRISK2 = 10–19%		QRISK2≥20%	
	No LLT^a^	Statins	No LLT	Statins	No LLT	Statins
Total	322	3	108,703	10,822	98,900	25,559
Women (%)	322 (100%)	3 (100%)	93,010 (86%)	9,928 (92%)	23,626 (24%)	9,570 (37%)
Men (%)	0 (0%)	0 (0%)	15,693 (14%)	894 (8%)	75,274 (76%)	15,989 (63%)
Born 1936–40 (%)	57 (18%)	3 (100%)	27,825 (26%)	6,887 (64%)	23,817 (24%)	17,132 (67%)
Born 1931–35 (%)	116 (36%)	0 (0%)	34,003 (31%)	3,264 (30%)	32,786 (33%)	7,363 (29%)
Born 1920–30 (%)	149 (46%)	0 (0%)	46,875 (43%)	671 (6%)	42,297 (43%)	1,064 (4%)
Townsend score • 1 least deprived (%)	211 (66%)	3 (100%)	37,455 (34%)	3,912 (36%)	25,212 (25%)	7,296 (29%)
• 2 (%)	83 (26%)	0 (0%)	28,616 (26%)	2,926 (27%)	22,215 (22%)	6,043 (24%)
• 3 (%)	24 (7%)	0 (0%)	20,804 (19%)	2,066 (19%)	19,945 (20%)	5,113 (20%)
• 4 (%)	4 (1%)	0 (0%)	15,032 (14%)	1,432 (13%)	18,198 (18%)	4,228 (17%)
• 5 most deprived (%)	0 (0%)	0 (0%)	6,796 (6%)	486 (4%)	13,330 (13%)	2,879 (11%)
Family history of cardiovascular disease (%)	0 (0%)	0 (0%)	206 (0%)	55 (1%)	543 (1%)	390 (2%)
Chronic kidney disease (%)	0 (0%)	0 (0%)	309 (0%)	184 (2%)	1,776 (2%)	2,948 (12%)
Diabetes (%)	0 (0%)	0 (0%)	4 (0%)	8 (0%)	8,384 (8%)	10,075 (39%)
Hypercholesterolaemia (%)	1 (0%)	0 (0%)	20,407 (19%)	5,706 (53%)	28,874 (29%)	12,237 (48%)
Treated hypertension (%)	0 (0%)	0 (0%)	22,742 (21%)	5,977 (55%)	36,534 (37%)	18,730 (73%)
Systolic blood pressure (sd)^b^	128 (18)	131 (40)	140 (17)	137 (14)	146 (18)	141 (16)
Ex-smoker (%)^b^	13 (4%)	0 (0%)	18,163 (17%)	2,132 (20%)	27,565 (28%)	9,607 (38%)
Smoker (%)^b^	10 (3%)	0 (0%)	5,501 (5%)	101 (1%)	30,799 (31%)	4,604 (18%)
Body mass index (sd)^b^	25 (4)	28 (6)	26 (5)	27 (5)	26 (4)	29 (5)

The number of participants in each risk-treatment group is the mean across ten imputed datasets.

^a^ lipid-lowering drugs ^b^ mean values across ten imputed datasets.

**Table 4:** Baseline characteristics of the 75-year old cohort.

	QRISK2 = 10–19%		QRISK2≥20%	
	No LLT	Statins	No LLT	Statins
Total	13,685	661	142,521	34,743
Women (%)	13,684 (100%)	661 (100%)	78,799 (55%)	19,566 (56%)
Men (%)	1 (0%)	0 (0%)	63,722 (45%)	15,177 (44%)
Born 1936–40 (%)	4,236 (31%)	500 (76%)	41,229 (29%)	25,690 (74%)
Born 1931–35 (%)	4,761 (35%)	139 (21%)	50,300 (35%)	8,171 (24%)
Born 1920–30 (%)	4,688 (34%)	22 (3%)	50,992 (36%)	882 (3%)
Townsend score • 1 least deprived (%)	5,736 (42%)	303 (46%)	39,371 (28%)	10,551 (30%)
• 2 (%)	3,907 (29%)	187 (28%)	33,866 (24%)	8,433 (24%)
• 3 (%)	2,491 (18%)	115 (17%)	28,856 (20%)	6,924 (20%)
• 4 (%)	1,295 (9%)	43 (7%)	24,836 (17%)	5,456 (16%)
• 5 most deprived (%)	256 (2%)	13 (2%)	15,592 (11%)	3,379 (10%)
Family history of cardiovascular disease (%)	5 (0%)	2 (0%)	531 (0%)	406 (1%)
Chronic kidney disease (%)	0 (0%)	0 (0%)	3,186 (2%)	5,172 (15%)
Diabetes (%)	0 (0%)	0 (0%)	7,039 (5%)	10,023 (29%)
Hypercholesterolaemia (%)	48 (0%)	12 (2%)	37,587 (26%)	16,417 (47%)
Treated hypertension (%)	34 (0%)	11 (2%)	55,086 (39%)	25,746 (74%)
Systolic blood pressure (sd)^b^	133 (15)	128 (12)	146 (18)	140 (15)
Ex-smoker (%)^b^	637 (5%)	32 (5%)	35,573 (25%)	11,692 (34%)
Smoker (%)^b^	164 (1%)	1 (0%)	22,577 (16%)	3,523 (10%)
Body mass index (sd)^b^	25 (4)	26 (4)	26 (4)	28 (5)

The number of participants in each risk-treatment group is the mean across ten imputed datasets. All participants aged 75 had a QRISK2 of 10% or higher. ^a^ lipid-lowering drugs ^b^ mean values across ten imputed datasets.

Prescription of statins increased considerably over the past 25 years (S1 Fig). Still only 45% of the participants with a cardiovascular risk of ≥20% were prescribed statins in 2010. Given risk group and calendar year, statins were prescribed less in men and in older participants (Tables 1–4 and S1 Fig, respectively). Given age-risk group, 50% of the therapy durations started between one and four years prior to the cohort’s age, 75% under six years, and only 10% more than eight years (S2 Fig). For the majority (87–93%) of these participants, the most recent prescription prior to the cohort’s age was within the last year. Adherence of treatment arm was ascertained for patients who were observed in multiple cohorts. Assuming that patients who were lost to follow-up stayed in the initial treatment arm, 88–98% of the cases and 78–92% of the controls never switched treatment arm (S7 Table). Five percent of the patients changed treatment arms multiple times.

**Fig 2:**
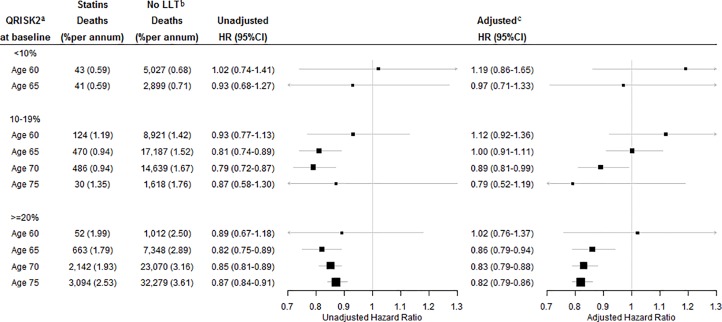
Unadjusted and adjusted effects of statin prescription on the hazard of mortality by age and cardiovascular risk group. ^a^ 10-year risk of a first cardiovascular event. ^b^ lipid-lowering therapy. ^c^ adjusted for sex, year of birth, socioeconomic status, diabetes, hypercholesterolaemia, blood pressure regulating drugs, body mass index, smoking status, and general practice.

In participants with a QRISK2 score less than 10%, statin prescription was not associated with reduced mortality at any age (adjusted hazard ratio, Fig 2). In those with a QRISK2 score of 10–19%, statin prescription at any time before age 65 was not associated with reduced mortality, but statin prescription for those aged 70 or 75 at baseline was associated with a hazard of mortality of 0.89 (95% CI 0.81–0.99) and 0.79 (95% CI 0.52–1.19), respectively. In those with a QRISK2 score ≥20%, statin prescription before age 60 was not associated with reduced mortality, but statin prescription for those aged 65, 70 or 75 at baseline was associated with a hazard of mortality of 0.86 (95% CI 0.79–0.94), 0.83 (95% CI 0.79–0.88), and 0.82 (95% CI 0.79–0.86), respectively. This translates to an increase in life of 1.5 (95% CI 0.6–2.4), 1.9 (95% CI 1.3–2.4), and 2.0 (95% CI 1.5–2.4) years, respectively, compared to participants without statin prescription by those ages. There were no interactions found with statin prescription. This means that the survival benefit associated with statin prescription was the same for various subgroups of patients such as men and women.

The adjusted effects in the complete case analysis had similar results and performance as the adjusted effects estimated on both complete and incomplete records (S3 Fig). The complete case analyses estimated 3–5% greater survival benefit from statin prescription, which could probably be explained by the fact that completeness of medical records was associated with ill health. The complete case models explained 11% of survival differentials, had 63% concordance between the hazard of mortality and survival time, and overestimated the coefficients by 5%.

## Discussion

This large population-based cohort study estimated the hazard of mortality associated with statin prescription for groups with <10%, 10–19%, or ≥20% risk of a first cardiovascular event in the next ten years, using QRISK2 over almost 25 years. It shows that there was no mortality reduction associated with statin prescription in the 60-year old cohort, and in participants at less than 10% risk aged 65, 70, or 75 at baseline. Participants aged 70 or 75 at baseline and who were at moderate risk (QRISK2 score 10–19%) showed uncertain benefit associated with statin prescription. Participants aged 65, 70, or 75 at baseline and who were at high risk (QRISK2 score ≥20%) had reduced mortality associated with statin prescription. The hazard ratios reduce with increasing age, with the greatest benefit seen in the oldest cohort. In keeping with previous research, there was no difference between men and women in mortality associated with statins [37].

### Strengths and limitations

This study used routinely collected primary care data that were representative of the UK population and widely available. The large sample size included a great number of participants aged over 80 and many people at low risk of a cardiovascular event, with almost 25 years of follow-up data. The 10-year risk of a first cardiovascular event was calculated by QRISK2, which is recommended by NICE and widely used in routine practice, and broadly comparable to other widely used risk assessment tools such as SCORE and PCE. The use of all-cause mortality, meant that the overall effect of statin prescription on mortality could be assessed as opposed to estimating the possible shift of the hazard of mortality from one medical condition to another. Estimating the effect of statin prescription by age group meant that age-group specific recommendations could be given.

The analysis was performed on an intention to treat basis to more accurately assess the effect of routine current practice in the general population. THIN had information on prescription of drugs, and not on dispensing and intake of them. This means that the actual statin uptake could be lower than THIN records indicated. The analysis did not include the duration of therapy as a possible predictor of survival. As the majority of the patients had started statin therapy within the six years prior to each key age for each risk group, it seems more likely that the benefit of statins is age-dependant rather than duration-dependant. In other words, it is unlikely that the younger participants did not experience a survival benefit from statins due to a shorter duration of therapy. Due to limitations of the data, the QRISK2 score could only be approximated. Missing data are described in the methods section and S5 Table. Although there were missing data, sensitivity analyses showed that it was unlikely they influenced the results.

Limitations of a prevalence-user (rather than new-user) study design are that bias might be introduced when the exposure’s effect on the outcome varies by time and alters other health indicators [38]. As the hazard of mortality associated with statin prescription did not differ by time, it is unlikely that underascertainment of deaths between the time statin therapy is initiated and the target age is reached would have biased the results.

Randomised controlled trials are rightly considered to be a stronger form of evidence than cohort studies, primarily because randomisation is designed to ensure that potential confounders are evenly distributed between control and intervention groups. A limitation of using routinely collected observational data to estimate the effects of interventions is that the results might be affected by unexplained confounding. This was minimised by stratifying by cardiovascular risk group and age, and by controlling in the regression models for a wide range of potential confounders. Despite this, we cannot completely exclude residual confounding by indication. As this is an observational study, both sick-user bias and healthy-user bias cannot be excluded. Sick-user bias may arise where at a given QRISK2 score, general practitioners recognised people who were at greater risk and consequently prescribed them statins. This bias would have led to an underestimation of the survival benefit of statin prescription. Healthy-user bias would arise where at a given QRISK2 score, patients who were more proactive about their health, were more likely to be treated with a statin [38–39]. This bias would have led to an overestimation of the survival benefit of statin prescription. We believe that both biases were mitigated by inclusion of co-morbidities, lifestyle factors and socioeconomic status in our models but cannot be excluded.

These findings reinforce the considerable benefits of statin treatment in high-risk groups (where we found substantial and important undertreatment) reported by the extremely influential CTT meta-analysis [5], but clearly differ from the CTT results for those at low risk. Where we found no benefit, despite widespread treatment of participants (particularly women) at low risk, the CTT reported an overall reduction in all-cause mortality (rate ratio 0.91) in participants without a history of cardiovascular disease over 2–5 years, and concluded that statins ‘are effective for people with a 5-year risk of major vascular events lower than 10%’ [5]. A more recent review reiterated the well-known methodological limitations of observational studies of treatment [40], which we agree with and have discussed above. However, the use of statins in primary prevention remains controversial and contested, perhaps at least in part due to the limitations of RCTs discussed in the introduction, including lack of generalisability due to strict inclusion criteria, lack of comparability with clinical risk scores such as QRISK2 due to trials use of observed events as a comparator (the CTT trial 5-year risk of major vascular events lower than 10% is hard to compare to a general population QRISK2 score of 10% and is not equivalent), the small number of older patients in trials, and the relatively short follow-up time of 4–5 years in trial (compared with 6–12 years in the age cohorts studied here), and perhaps most importantly, the concerns that anonymised individual patient data from statins trials have still not been made available for independent scrutiny, and remain under the control of a single group of respected researchers, whilst statins are among the most widely prescribed drugs globally [7, 41]. Large scale observational studies of the effects of statins in everyday diverse clinical practice over many years are an under-explored source of information on the effects of statins on mortality. No data source is perfect and there are well rehearsed uncertainties and unanswered questions arising from both observational and trial data (set out in the introduction), and we believe that this analysis of cohort data fills in some of the gaps and provides an important new source of information on the possible effects of statins in routine practice.

### Recommendations

Further research is needed on the effects of statins over the long term for younger patients at low risk of a future first cardiovascular event. These findings suggest that the current internationally recommended thresholds for statin therapy for primary prevention of cardiovascular disease in routine practice are too low, and may lead to overtreatment, particularly of people under 60 and at low (<10%) risk. The recent revision of guidelines to extend treatment to younger and lower risk groups may need to be reconsidered, and clinicians may want to use this new information when discussing the risks and benefits of statin initiation with their patients.

## Supporting Information

S1 Fig Statin prescription rate prior to target age^a^, by QRISK2^b^ from 1987–2011. ^a^ Prescription rate in a given year was the percentage of participants who turned the cohort’s age in that year and were prescribed statins prior to that age. ^b^ Mean 10-year risk of a first cardiovascular event across ten imputed datasets.
(TIF)

S2 Fig Prevalence duration of statin therapy given prescription prior to target age by QRISK2 group. The number of participants in each risk group is the mean across ten imputed datasets.
(TIF)

S3 Fig Complete case analysis: unadjusted and adjusted effects of statin prescription on the hazard of mortality. ^a^ 10-year risk of a first cardiovascular event. ^b^ Lipid-lowering therapy. ^c^ Adjusted for sex, year of birth, socioeconomic status, diabetes, hypercholesterolaemia, blood pressure regulating drugs, body mass index, smoking status, and general practice.
(TIF)

S1 Table Missing data in QRISK2 algorithm. (DOCX)

S2 Table Coding of variables. ^a^ Latest reading before entering the study, which was at the 1st of January of the year the participant turned the cohort’s age. ^b^ First category functioned as the baseline.
(DOCX)

S3 Table Coding of socioeconomic status Mosaic. (DOCX)

S4 Table Characteristics of participants with complete and incomplete^a^ medical records. ^a^ Missing record in systolic blood pressure, smoking status, or body mass index. ^b^ Mean (standard deviation) 10-year risk of a first cardiovascular event across ten imputed datasets.
(DOCX)

S5 Table Extra information about statistical analyses. (DOCX)

S6 Table Distribution of known and imputed values^a^ of variables with missing data. ^a^ Mean across ten imputed datasets.
(DOCX)

S7 Table Cases and controls staying in initial treatment arm during follow-up. Adherence of treatment arm in participants who were observed in multiple cohorts. Participants were not observed in an older age cohort when one of the following events happened: cardiovascular event, death, transfer to other general practice, or end of study. It was assumed that participants lost to follow-up stayed in the initial treatment arm.
(DOCX)
