# Supplementary figures and images for: Survival Benefits of Statins for Primary Prevention: A Cohort Study
Source: PLoS One. 2016 Nov 18;11(11):e0166847. doi: 10.1371/journal.pone.0166847 (PMC5115824; doi:10.1371/journal.pone.0166847)

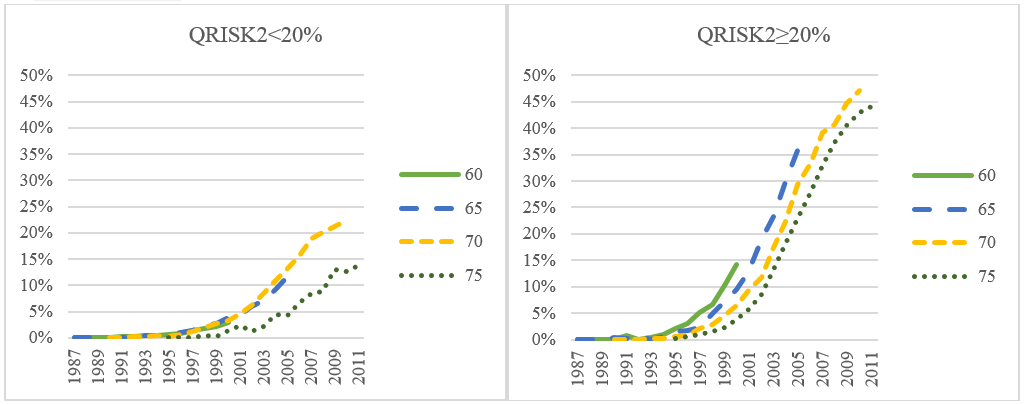

Supplement: S1 Fig — a Prescription rate in a given year was the percentage of participants who turned the cohort’s age in that year and were prescribed statins prior to that age. b Mean 10-year risk of a first cardiovascular event across ten imputed datasets. (TIF) [file pone.0166847.s001.tif]

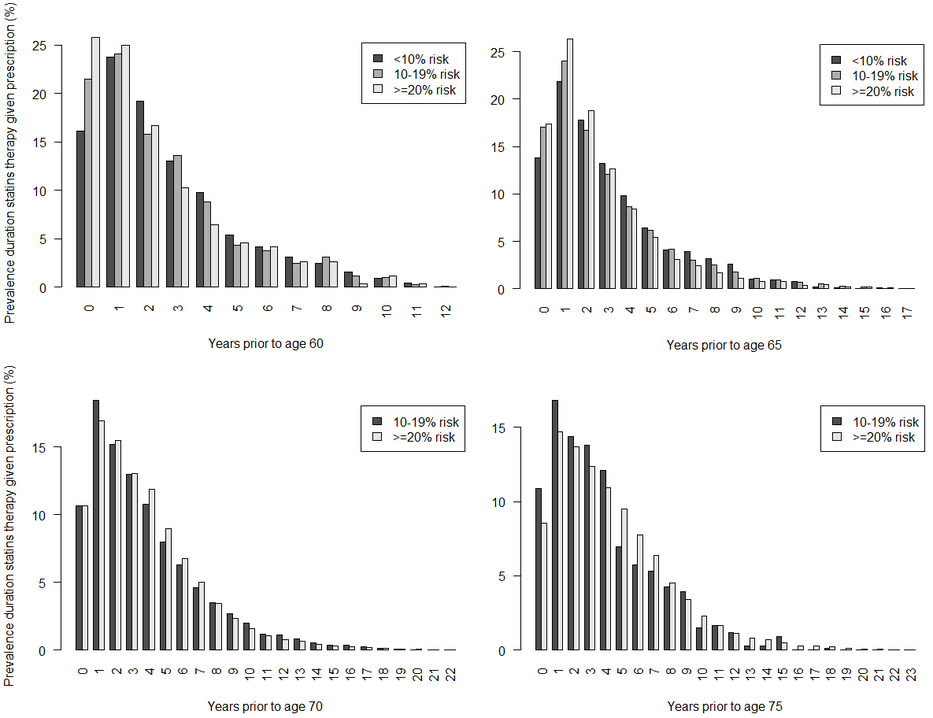

Supplement: S2 Fig — The number of participants in each risk group is the mean across ten imputed datasets. (TIF) [file pone.0166847.s002.tif]

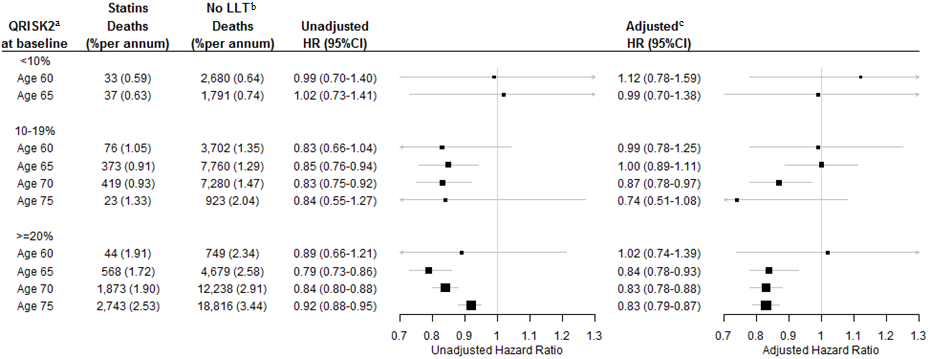

Supplement: S3 Fig — a 10-year risk of a first cardiovascular event. b Lipid-lowering therapy. c Adjusted for sex, year of birth, socioeconomic status, diabetes, hypercholesterolaemia, blood pressure regulating drugs, body mass index, smoking status, and general practice. (TIF) [file pone.0166847.s003.tif]
