# Supplementary material for: Survival Benefits of Statins for Primary Prevention: A Cohort Study
Source: PLoS One. 2016 Nov 18;11(11):e0166847. doi: 10.1371/journal.pone.0166847 (PMC5115824; doi:10.1371/journal.pone.0166847)
Supplement: S1 Table — (DOCX) [file pone.0166847.s004.docx]

**S1 Table.**

| **Variables in QRISK2** | **Available in studied age cohorts** |
| --- | --- |
| Self-assigned ethnicity (white/not recorded, Indian, Pakistani, Bangladeshi, other Asian, black African, black Caribbean, Chinese, other including mixed) | No: excluded |
| Age (years) | Yes |
| Sex (males versus females) | Yes |
| Smoking status (no/ex/light/moderate/heavy) | No: classified smokers as moderate smoker |
| Systolic blood pressure (continuous) | Yes |
| Ratio of total serum cholesterol/high density lipoprotein cholesterol (continuous) | No: substituted based on hypercholesterolaemia (HCL) diagnosis. Participants with no HCL diagnosis, are ascribed a value of 4. Participants with a HCL diagnosis, are ascribed a value of 5. |
| Body mass index (continuous) | Yes |
| Family history of coronary heart disease in first degree relative under 60 years (no/yes) | No: substituted by family history of cardiovascular disease |
| Townsend deprivation score (continuous) | No: used corresponding median value for quintiles: -3.15, -2.17, -1.05, 0.84, and 4.51 |
| Treated hypertension (diagnosis of hypertension and at least one current prescription of at least one antihypertensive agent) | Yes |
| Rheumatoid arthritis (no/yes) | No: excluded |
| Chronic renal disease (no/yes) | Yes |
| Diabetes (no/type1/type2) | No: classified diabetics as type two |
| Atrial fibrillation (no/yes) | No: excluded |
