# Supplementary material for: Survival Benefits of Statins for Primary Prevention: A Cohort Study
Source: PLoS One. 2016 Nov 18;11(11):e0166847. doi: 10.1371/journal.pone.0166847 (PMC5115824; doi:10.1371/journal.pone.0166847)
Supplement: S2 Table — a Latest reading before entering the study, which was at the 1st of January of the year the participant turned the cohort’s age. b First category functioned as the baseline. (DOCX) [file pone.0166847.s005.docx]

**S2 Table.**

| **Variables^a^** | **Coding^b^** |
| --- | --- |
| Body mass index (BMI), calculated as:(weight in kg)/(height in m)² | Under/normal weight: <25 / overweight: 25-30 / obese: ≥30 |
| Blood pressure regulating drugs prescription includes: beta-adrenoceptor blocking drugs, thiazides and related diuretics, adrenergic neurone blocking drugs, alpha-adrenoceptor blocking drugs, angiotensin-converting enzyme inhibitors, angiotensin-II receptor antagonists, centrally acting antihypertensive drugs, drugs affecting the renin-angiotensin system, drugs related to hypertension and heart failure, renin inhibitors, vasodilator antihypertensive drugs, and calcium-channel blockers | No/yes |
| Chronic kidney disease stages 3-5 diagnosis (GFR<60mL/min) | No/yes |
| Diabetes mellitus | No/yes |
| Hypercholesterolaemia or a total cholesterol reading of greater than 5mmol/L | No/yes |
| Hypertension diagnosis or a blood pressure of greater than 90/140mmHg | No/yes |
| Lipid-lowering therapy prescription includes a type of statin or one of the following: colesevelam, colestipol, colestyramine, ezetimibe, bezafibrate, ciprofibrate, clofibrate, fenofibrate, gemfibrozil, acipimox, nicotinic acid, and omega-3-triglycerides including other esters and acids |  |
| Sex | Female/male |
| Smoking status | No/ex/yes |
| Socioeconomic status measured by Mosaic, a consumer classification that captures demographics, lifestyles, and behaviours of people on postcode level | Ten categories, see Table S3 in the Supplemental File |
| Statin prescription includes: atorvastatin, cerivastatin, fluvastatin, pravastatin, rosuvastatin, or simvastatin | No/yes |
| Year of birth category | 1920-25 / 1926-29 / 1930-35 / 1936-40 |
