# Supplementary material for: Survival Benefits of Statins for Primary Prevention: A Cohort Study
Source: PLoS One. 2016 Nov 18;11(11):e0166847. doi: 10.1371/journal.pone.0166847 (PMC5115824; doi:10.1371/journal.pone.0166847)
Supplement: S3 Table — (DOCX) [file pone.0166847.s006.docx]

**S3 Table.**

| **Category** | **Description [26]** |
| --- | --- |
| Alpha territory | Most wealthy and inﬂuential individuals (reference category) |
| Professional rewards | Executive and managerial classes |
| Rural solitude | People who live in small villages |
| Small town diversity | People who live in medium sized and smaller towns |
| Careers and kids | Young couples, married or living with their partner |
| New homemakers | Neighbourhoods containing mostly houses that were built in the last ﬁve years |
| Ex-council community | Neighbourhoods populated by people who are practical and enterprising |
| Claimant cultures | Some of the most disadvantaged people |
| Upper floor living | People who are on limited incomes |
| Other | People living in neighbourhoods not mentioned above |
