# Supplementary material for: Survival Benefits of Statins for Primary Prevention: A Cohort Study
Source: PLoS One. 2016 Nov 18;11(11):e0166847. doi: 10.1371/journal.pone.0166847 (PMC5115824; doi:10.1371/journal.pone.0166847)
Supplement: S4 Table — a Missing record in systolic blood pressure, smoking status, or body mass index. b Mean (standard deviation) 10-year risk of a first cardiovascular event across ten imputed datasets. (DOCX) [file pone.0166847.s007.docx]

**S4 Table.**

| **Cohort** | **Records** | **Year of birth** | **Size** | **Death/1,000 Person-Years** | **Lipid-lowering therapy** | **QRISK2^b^ (sd)** |
| --- | --- | --- | --- | --- | --- | --- |
| Age 60 | Complete | 1936-40 | 45,807 | 8.9 | 1,734 (3.8%) | 10.6 (5.3) |
|  |  | 1930-35 | 18,104 | 11.7 | 324 (1.8%) | 10.4 (5.0) |
|  | Incomplete | 1936-40 | 29,572 | 9.8 | 495 (1.7%) | 10.4 (3.7) |
|  |  | 1930-35 | 25,217 | 13.0 | 196 (0.8%) | 10.2 (3.8) |
|  | Total |  | 118,700 | 10.6 | 2,749 (2.3%) | 10.4 (4.6) |
| Age 65 | Complete | 1936-40 | 66,764 | 10.7 | 9,884 (14.8%) | 15.9 (6.7) |
|  |  | 1931-35 | 40,060 | 14.1 | 1,986 (5.0%) | 15.2 (6.3) |
|  |  | 1925-30 | 15,998 | 20.0 | 299 (1.9%) | 15.1 (6.1) |
|  | Incomplete | 1936-40 | 28,558 | 11.6 | 1,345 (4.7%) | 14.9 (4.7) |
|  |  | 1931-35 | 24,872 | 16.0 | 583 (2.3%) | 14.6 (4.7) |
|  |  | 1925-30 | 23,322 | 21.6 | 201 (0.9%) | 14.5 (4.7) |
|  | Total |  | 199,574 | 15.2 | 14,298 (7.2%) | 15.2 (5.9) |
| Age 70 | Complete | 1936-40 | 66,733 | 12.7 | 24,235 (36.3%) | 22.5 (7.7) |
|  |  | 1931-35 | 55,423 | 17.0 | 9,970 (18.0%) | 22.0 (7.4) |
|  |  | 1920-30 | 46,111 | 26.3 | 1,849 (4.0%) | 21.2 (7.1) |
|  | Incomplete | 1936-40 | 10,292 | 13.6 | 1,091 (10.6%) | 19.9 (5.4) |
|  |  | 1931-35 | 22,855 | 19.3 | 1,403 (6.1%) | 20.1 (5.5) |
|  |  | 1920-30 | 45,735 | 29.8 | 676 (1.5%) | 19.7 (5.3) |
|  | Total |  | 247,149 | 22.8 | 39,224 (15.9%) | 21.3 (7.0) |
| Age 75 | Complete | 1931-36 | 64,358 | 20.4 | 26,585 (41.3%) | 30.4 (8.2) |
|  |  | 1926-30 | 43,947 | 29.1 | 7,761 (17.7%) | 29.7 (7.9) |
|  |  | 1920-25 | 29,948 | 40.3 | 921 (3.1%) | 28.6 (7.6) |
|  | Incomplete | 1931-36 | 8,796 | 22.7 | 1,104 (12.6%) | 26.5 (5.8) |
|  |  | 1926-30 | 20,024 | 32.5 | 1,149 (5.7%) | 26.8 (5.7) |
|  |  | 1920-25 | 27,012 | 41.8 | 359 (1.3%) | 26.6 (5.5) |
|  | Total |  | 194,085 | 33.4 | 37,879 (19.5%) | 28.9 (7.5) |
