# Supplementary material for: Survival Benefits of Statins for Primary Prevention: A Cohort Study
Source: PLoS One. 2016 Nov 18;11(11):e0166847. doi: 10.1371/journal.pone.0166847 (PMC5115824; doi:10.1371/journal.pone.0166847)
Supplement: S5 Table — (DOCX) [file pone.0166847.s008.docx]

**S5 Table.**

Values were imputed for QRISK2, systolic blood pressure, body mass index, and smoking status. The joint imputation model that was multilevel on patient and practice level consisted of all factors included in the QRISK2 calculation and the outcome variable of the analysis model which was time to death. The reason why QRISK2 score was imputed instead of calculated based on the imputed values (i.e. passive imputed) is the same reason why interaction effects are imputed, which is to ensure that the imputed values are consistent with the analysis model [30]. The imputation process had a length of 500 iterations from which ten imputed datasets were derived. This process was run in REALCOM-Imputation software developed by the Centre for Multilevel Modelling based at the University of Bristol. The effects of statin prescription on survival were estimated on the imputed datasets. The results were combined using Rubin’s rules to deal with the uncertainty caused by missing data [29].

The number of years gained or lost due to statin prescription were calculated using the estimated hazard ratios by the Cox’s regression: 10*ln(hazard ratio) [32]. This is independent of age given that the baseline hazard of mortality increases exponentially with age and that the proportional hazards assumption holds. The first assumption was checked by plotting the logarithm of the baseline hazard against follow-up time. The assumption holds when there is a horizontal line. The proportional hazards assumption was checked by the Grambsch and Therneau’s test [33]. When there was a significant violation (p<.01), the variable’s effect on the hazard of mortality was made time-variant.

A model’s overall performance was quantified by Royston’s R², which calculates the amount of explained variation in survival time [34]. A model’s discrimination was quantified by Harrell’s Concordance, which computes the percentage of agreement between the risk score and survival time for all combinations of two selected subjects [35]. Survival models typically have a concordance of 60 to 70%. External validation was assessed by the shrinkage slope, which quantifies the percentage by which the model overestimates the effects on the hazard of mortality. This was calculated by a ten-fold cross-validation [36].
