# Supplementary material for: Survival Benefits of Statins for Primary Prevention: A Cohort Study
Source: PLoS One. 2016 Nov 18;11(11):e0166847. doi: 10.1371/journal.pone.0166847 (PMC5115824; doi:10.1371/journal.pone.0166847)
Supplement: S6 Table — a Mean across ten imputed datasets. (DOCX) [file pone.0166847.s009.docx]

**S6** **Table.**

|  | **Age 60** | | **Age 65** | | **Age 70** | | **Age 75** | |
| --- | --- | --- | --- | --- | --- | --- | --- | --- |
| **Factors** | **Known** | **Imputed** | **Known** | **Imputed** | **Known** | **Imputed** | **Known** | **Imputed** |
| Smoker status | 81,973 (69.6%) | 35,851 (30.4%) | 155,100 (77.7%) | 44,474 (22.3%) | 204,304 (82.7%) | 42,845 (17.3%) | 166,947 (86.0%) | 27,138 (14.0%) |
| Ex-smoker | 12.9% | 13.0% | 18.3% | 19.9% | 23.4% | 24.4% | 24.8% | 26.5% |
| Smoker | 25.5% | 28.1% | 21.5% | 23.3% | 16.5% | 18.0% | 13.4% | 15.0% |
| BMI | 74,153 (62.9%) | 43,671 (37.1%) | 141,223 (70.8%) | 58,351 (29.2%) | 186,040 (75.3%) | 61,109 (24.7%) | 150,555 (77.6%) | 43,530 (22.4%) |
| BMI (sd) | 26.4 (4.4) | 26.2 (4.4) | 26.6 (4.5) | 26.2 (4.4) | 26.7 (4.6) | 26.0 (4.5) | 26.5 (4.6) | 25.9 (4.5) |
| SBP | 84,567 (71.8%) | 33,257 (28.2%) | 153,810 (77.7%) | 44,046 (22.3%) | 200,881 (82.2%) | 43,428 (17.8%) | 163,361 (85.3%) | 28,249 (14.7%) |
| SBP (sd) | 139.1 (18.4) | 136.4 (17.7) | 141.6 (17.9) | 138.8 (17.6) | 142.4 (17.5) | 142.0 (17.5) | 143.7 (17.7) | 143.1 (17.7) |
| QRISK2 | 63,271 (53.7%) | 54,553 (46.3%) | 122,822 (61.5%) | 76,752 (38.5%) | 168,267 (68.1%) | 78,882 (31.9%) | 138,253 (71.2%) | 55,832 (28.8%) |
| QRISK2 (sd) | 10.5 (5.2) | 10.3 (4.9) | 15.6 (6.5) | 14.7 (6.3) | 22.0 (7.5) | 19.8 (7.2) | 29.8 (8.0) | 26.7 (7.9) |
