# Supplementary material for: Survival Benefits of Statins for Primary Prevention: A Cohort Study
Source: PLoS One. 2016 Nov 18;11(11):e0166847. doi: 10.1371/journal.pone.0166847 (PMC5115824; doi:10.1371/journal.pone.0166847)
Supplement: S7 Table — Adherence of treatment arm in participants who were observed in multiple cohorts. Participants were not observed in an older age cohort when one of the following events happened: cardiovascular event, death, transfer to other general practice, or end of study. It was assumed that participants lost to follow-up stayed in the initial treatment arm. (DOCX) [file pone.0166847.s010.docx]

**S7** **Table.**

| **Cohort** | **Cases** | **Controls** |
| --- | --- | --- |
| Age 60 | 1,469/1,664 (88%) | 91,691/117,036 (78%) |
| Age 65 | 3,935/4,278 (92%) | 82,399/98,097 (84%) |
| Age 70 | 4,560/4,673 (98%) | 78,565/85,961 (92%) |
